# High-resolution imaging and computational analysis of haematopoietic cell dynamics *in vivo*

**DOI:** 10.1038/ncomms12169

**Published:** 2016-07-18

**Authors:** Claire S. Koechlein, Jeffrey R. Harris, Timothy K. Lee, Joi Weeks, Raymond G. Fox, Bryan Zimdahl, Takahiro Ito, Allen Blevins, Seung-Hye Jung, John P. Chute, Amit Chourasia, Markus W. Covert, Tannishtha Reya

**Affiliations:** 1 Departments of Pharmacology and Medicine, School of Medicine, University of California, San Diego, La Jolla, California 92093, USA; 2 Sanford Consortium for Regenerative Medicine, La Jolla, California 92037, USA; 3 Department of Pharmacology and Cancer Biology, Duke University Medical Center, Durham, North Carolina 27710, USA; 4 Department of Bioengineering, Stanford University, Stanford, California 94305, USA; 5 Division of Cellular Therapy, Duke University Medical Center, Durham, North Carolina 27710, USA; 6 Division of Hematology Oncology, Department of Medicine, University of California, Los Angeles, Los Angeles, California 90095, USA; 7 San Diego Supercomputer Center, University of California, San Diego, La Jolla, California 92093, USA

## Abstract

Although we know a great deal about the phenotype and function of haematopoietic stem/progenitor cells, a major challenge has been mapping their dynamic behaviour within living systems. Here we describe a strategy to image cells *in vivo* with high spatial and temporal resolution, and quantify their interactions using a high-throughput computational approach. Using these tools, and a new Msi2 reporter model, we show that haematopoietic stem/progenitor cells display preferential spatial affinity for contacting the vascular niche, and a temporal affinity for making stable associations with these cells. These preferences are markedly diminished as cells mature, suggesting that programs that control differentiation state are key determinants of spatiotemporal behaviour, and thus dictate the signals a cell receives from specific microenvironmental domains. These collectively demonstrate that high-resolution imaging coupled with computational analysis can provide new biological insight, and may in the long term enable creation of a dynamic atlas of cells within their native microenvironment.

## Abstract (web summary)

It is difficult to image haematopoietic stem cells (HSC) in their niche. Here, the authors present a new high-throughput computational approach to visualise HSCs *in vivo* at a high spatial and temporal resolution and also use a Msi2-reporter to label endogenous HSCs and progenitors, enabling cell tracking

## Graphical abstract

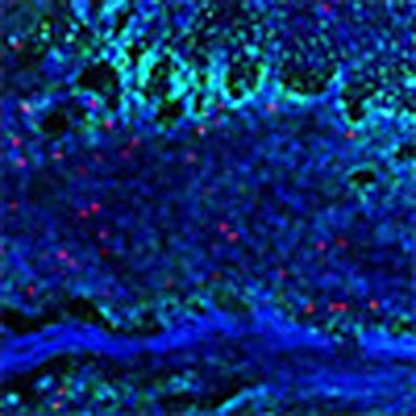

The haematopoietic system is responsible for generating all the cells of the blood and immune system. The development of fully mature cells from immature haematopoietic stem and progenitor cells occurs in a highly regulated manner within the bone marrow, the primary site of adult haematopoiesis^1^. Here cells integrate a multitude of soluble and cell contact-derived signals from their microenvironment or niche to achieve and maintain tissue homeostasis^2^,^3^,^4^, as well as to initiate regeneration in response to injury^5^. Defining the dynamic interactions of haematopoietic cells with the microenvironment over time and space is thus critically important to better understanding haematopoiesis.

Traditionally, studies of these interactions have been largely restricted to static analysis primarily due to limitations in imaging technology and tissue accessibility^6^,^7^,^8^,^9^,^10^,^11^,^12^. Of note, advances in the field have improved the utility of this approach. For example, in a recent study, optical clearing of the bone marrow permitted deep confocal imaging of haematopoietic cells and digital reconstruction of the marrow cavity^13^. However, the dynamic changes that occur as cells interact with components of the bone marrow microenvironment are not readily captured by these methods. To address this, several groups have used two-photon intravital imaging within the bone marrow cavity of the calvarium^14^,^15^,^16^ or the long bone^17^. While these studies have provided valuable new ways to visualize the haematopoietic compartment and to generate three-dimensional spatial models of the bone marrow microenvironment in living animals, there is a continued need for not only increasing spatiotemporal resolution but also a strategy to track endogenous cells without transplantation and a means by which the ‘big data' that is generated by such imaging approaches can be analysed to reveal new biological patterns. This would enable us to better map the interactions, signals and mechanisms that govern haematopoietic cell behaviour and function *in vivo*, and thereby understand how this can fail in disease and degeneration.

To address this need, we have developed an approach that allows real-time imaging of haematopoietic cells in the context of their living microenvironment with high spatial and temporal resolution. Notably, the resolution achieved has allowed us to build a new analytic tool that permits *in vivo* tracking of individual cells and their temporal and spatial behaviour relative to microenvironmental niches. In addition to tracking transplanted haematopoietic cells, we also tracked endogenous immature haematopoietic cells using a newly developed Musashi2 (Msi2) knock-in reporter mouse. This mouse reports endogenous expression of Musashi2 (reporter for Musashi2, REM2) with enhanced green fluorescence protein (eGFP)^18^. Because Msi2 is highly expressed within haematopoietic stem and progenitor cells^19^, Msi2GFP^bright^ expression faithfully marks an immature haematopoietic population, which can be dynamically tracked *in vivo*. This reporter mouse, in conjunction with high-resolution live imaging, makes it possible to dynamically track endogenous immature cells *in vivo*. Using these tools, we have identified spatial ‘hotspots' within the microenvironment: areas where haematopoietic stem and progenitor cells preferentially reside and interact. Specifically, we find that immature haematopoietic cells have a significant preference for being in stable contact with vascular domains, while differentiated cells make more short-term interactions and frequently shuttle between the vascular and endosteal domains. These suggest that differentiation state can control the spatiotemporal behaviour of haematopoietic cells and the programs that control cell fate also dictate the kinds of signals cells will be exposed to by the virtue of their localization in specific microdomains. These data show that high-resolution imaging coupled with an effective high-throughput computational approach can provide new biological insight into the dynamics of haematopoietic cells in their microenvironment, and can be used to establish a baseline to study the changes in haematopoietic cell interactions within the niche during regeneration and oncogenesis.

## Results

### Real-time imaging

**Figure 1:**
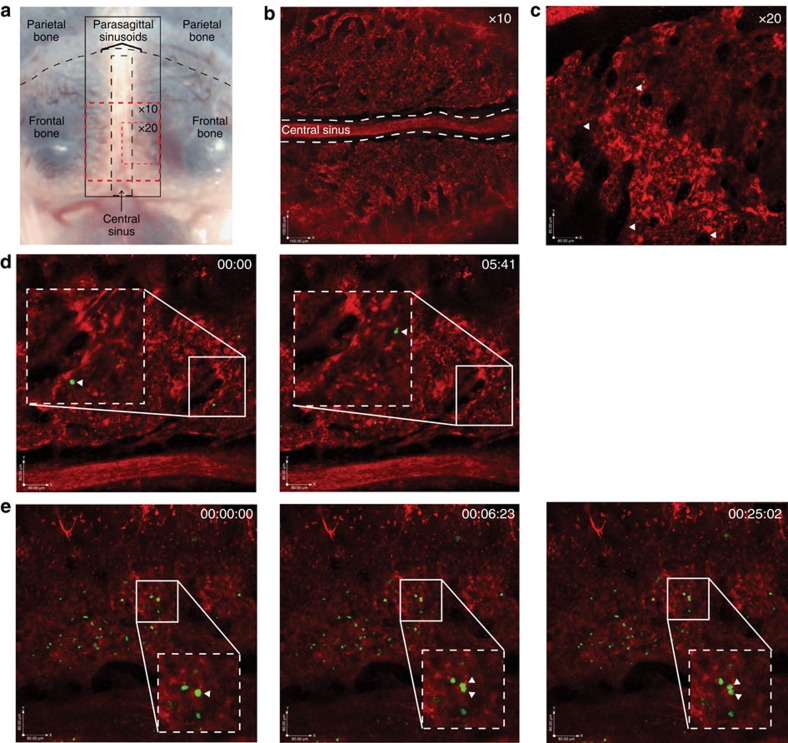
Real-time imaging. (**a**) Representative photo showing architecture of imaging area in mouse calvarium. Red-dashed boxes indicate representative areas imaged. Black-dashed box highlights the central sinus. Parasagittal sinusoids flank either side of the central sinus. (**b**) Representative × 10 image of transgenic dsRed mouse calvarium. White-dashed lines highlight the central sinus. Scale bar, 150 μm. (**c**) Representative × 20 image of dsRed bone marrow. Closed triangles depict transplanted Lin^−^GFP^+^ cells in the microenvironment. Scale bar, 80 μm. See Supplementary Movie 1. (**d**) Still image of a Lin^−^GFP^+^ haematopoietic cell (closed triangle) rolling along the vessel wall in a dsRed recipient mouse, shown are images taken at *t*=0 (left panel) and at end point *t*=05:41 (right panel). See Supplementary Movie 2. Inlay= × 1.5 zoom; scale bar, 80 μm. (**e**) Still image of a Lin^−^GFP^+^ haematopoietic cell in division. *t*=0: arrow identifies cell preparing to divide, *t*=6:23: arrows indicate cell in the midst of division and *t*=25:02: arrows indicate two daughter cells. See Supplementary Movie 3. Scale bar, 80 μm, box × 1.5 zoom of field.

To understand how haematopoietic stem and progenitor cells behave in living tissues, we developed a real-time imaging strategy to visualize cells in high-resolution over extended periods of time. We used fluorescent protein-expressing transgenic mice to observe the spatial orientation of the bone marrow cavity, and a typical confocal microscope to view inside the mouse calvarium (Fig. 1a)^20^. Mice were anaesthetized, their calvaria exposed and they were placed either inverted on an imaging apparatus or upright in a stereotactic device. Stabilization of animals was important for limiting breathing artefacts that can occur during an imaging session, and consistency of animal orientation was crucial for successful imaging of the same region (in the parasagittal sinusoids) between mice and between experiments over extended periods of time (Supplementary Fig. 1).

To highlight the features of the bone marrow microenvironment, mice with constitutive expression of dsRed under the control of a ubiquitous promoter were used^21^. This strategy provided a counterlabel and had a ‘backlighting' effect for visualizing microenvironment cells. A typical × 10 image of the bone marrow of a dsRed mouse is shown in Fig. 1b. When analysing haematopoietic cell movement, we used a higher magnification objective (× 20) to achieve greater spatial resolution (Fig. 1c). The use of transgenic mice expressing dsRed.T3 (ref. 22) to create a labelled microenvironment was particularly important in allowing tracking of haematopoietic cell encounters and associations with the niche at a single-cell level.

The high degree of temporal and spatial resolution allowed clear visualization of transplanted cells interacting for several minutes to several hours with specific niches. In these experiments, actin promoter-driven GFP^+^ haematopoietic progenitor cells, as defined by the absence of lineage markers (lineage negative or Lin^−^), were transplanted into dsRed recipient mice (Fig. 1d; Supplementary Movie 1). This strategy provided a way to distinguish associations that lasted short periods of time and those that were more stable, lasting several hours. Further, individual GFP^+^ cells could be tracked rolling/crawling along the endothelium within the calvarial marrow (Fig. 1d, arrows; Supplementary Movie 2). Finally, we were able to trace individual cells dividing in real time (Fig. 1e; Supplementary Movie 3), a testament to the single-cell resolution achieved in this system.

**Figure 2:**
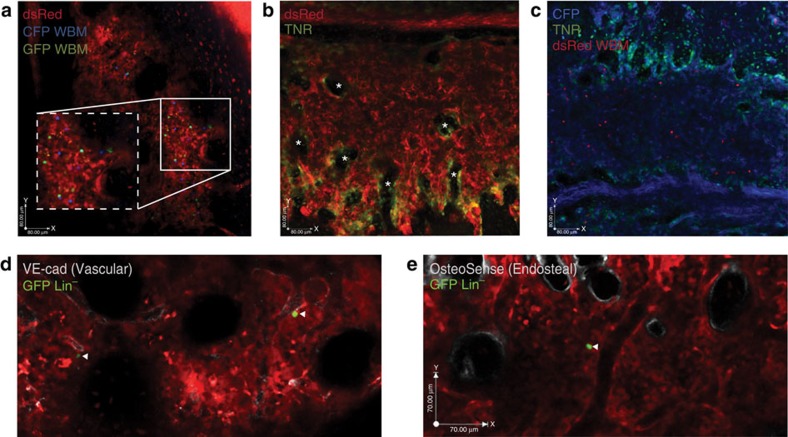
Multicolour analysis. (**a**) Representative three-colour analysis of a dsRed recipient transplanted with GFP^+^ and CFP^+^ whole bone marrow (WBM) cells. Corresponding movie shows a *z*-stack step through the marrow microenvironment (Supplementary Movie 4). Scale bar, 80 μm, box × 1.5 zoom of field. (**b**) Representative image of dsRed mouse crossed to TNR showing Notch signalling domains within the bone marrow microenvironment; green signal reflects the Notch reporter activity within the microenvironment (asterisks). (**c**) Representative image of dsRed^+^ WBM transplanted into TNR.CFP mouse. Green reflects the Notch reporter activity within the microenvironment (Supplementary Movie 5). Images **a**–**c** were obtained with a × 20 objective. Scale bar, 80 μm. (**d**,**e**) dsRed mice (red) transplanted with GFP^+^Lin^−^ cells (green) and co-labelled with conjugated probes to (**d**) endothelial cells (anti-VE-cadherin antibody, Supplementary Movie 6) and (**e**) the endosteal surface (OsteoSense, Supplementary Movie 7). Scale bar, 70 μm.

Different fluorescent proteins with multiple spectra were used to analyse distinct cell populations simultaneously. To test imaging in multiple colours, we transplanted bone marrow cells from GFP and cyan fluorescent protein (CFP) donors into dsRed recipients. As shown in the three-dimensional view of the recipient marrow, we could clearly distinguish both GFP^+^ and CFP^+^ cells within the dsRed backlit microenvironment (Fig. 2a; Supplementary Movie 4). The use of multiple colours enabled comparative imaging of cells from distinct genetic backgrounds within the same niche. We also tested whether we could monitor the signalling status of niche cells. To this end, we crossed actin-dsRed mice to transgenic notch reporter mice (TNR), in which GFP is predominantly expressed in cells responding to the Notch signalling^23^,^24^,^25^. Notch signalling was active in cells surrounding areas of bone and in the endosteal region within the microenvironment (Fig. 2b, asterisks). Further, the association of haematopoietic cells with Notch reporter^+^ cells could be visualized by transplanting dsRed haematopoietic cells into TNR mice crossed with CFP mice (Fig. 2c; Supplementary Movie 5). These data show that haematopoietic cells and their interactions with niche cells responsive to a specific signal can be traced at a single-cell level *in vivo*.

### Computational analysis

The ability to clearly assess niches in real-time coupled with the spatial and temporal resolution allowed us to begin to generate a map of haematopoietic cell associations with the niche in homeostasis. To maintain the most flexibility, we visualized elements of the environment using ectopically delivered antibodies and probes. Vascular endothelial cells and blood vessels were identified using anti-VE-cadherin antibodies and the *in vivo* probe angiosense, respectively (Fig. 2d; Supplementary Fig. 2a–b; Supplementary Movie 6), and the endosteal region was identified using the *in vivo* probe OsteoSense (Fig. 2e; Supplementary Fig. 2c–d; Supplementary Movie 7). Other potential niche cells, such as tissue macrophages, could also be visualized using this strategy (Supplementary Fig. 2e; Supplementary Movie 8), and may be of future interest.

**Figure 3:**
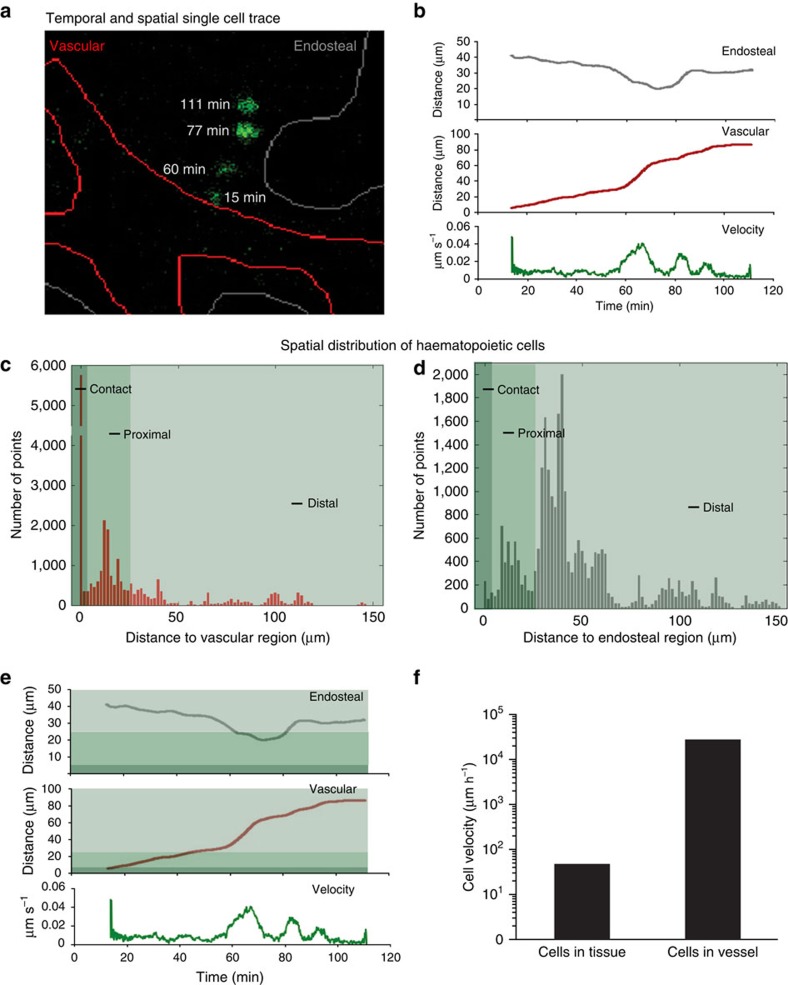
Computational image analysis of spatial dynamics. (**a**) Automated analysis enables tracking of individual cells (green) over time, and determination of the distance from the vascular (outlined in red) and endosteal (outlined in grey) regions. See Supplementary Movie 9. (**b**) For the same cell shown in **a**, the distance to the endosteal (grey line) and vascular (red line) regions as well as the cell velocity (green line) are shown. (**c**,**d**) Histograms containing the distance to the vascular (**c**) and endosteal (**d**) regions for all cells analysed at all time points assessed. Three ‘zones' were determined from these histograms: contact, proximal and distal. (**e**) The same data shown in **b** but with the three zones highlighted. (**f**) Average cellular velocity for all cells observed in the bone marrow (regardless of lineage status), compared with the average velocity of cells in a blood vessel.

The spatial location of GFP^+^ transplanted cells could be clearly viewed relative to the microdomain of interest (Fig. 2d,e, arrows). Beginning with the raw image set, our software automatically corrects for lateral drift between images, identifies individual cells and tracks the position of each cell over time using particle-tracking software (Fig. 3a; Supplementary Movie 9; see Methods). The program then records the *x* and *y* coordinates at each time point, as well as the distance travelled and cellular velocity. In addition, with defined endosteal and vascular microdomains (another input to the software), the program calculates the closest distance between these regions and each cell. For example, Fig. 3a shows how one cell, which initially localized close to a vascular (red) region, migrated over time towards an endosteal (grey) region. Figure 3b is a trace depicting the quantitative data derived using our software.

Using this approach, we wanted to determine whether there were characteristic distances at which haematopoietic cells interacted with specific regions of the microenvironment. Thus, we first plotted each cell's distance to the vasculature at every time point (Fig. 3c). The resulting histogram suggested that a significant amount of cellular time was spent within 5 μm of the vasculature, and, based on visual confirmation, was designated as ‘contact'. Interestingly, the region between 5 and 25 μm was also highly enriched in terms of cellular presence, and was designated as a ‘proximal' zone. Distances greater than 25 μm from a niche of interest were designated as a ‘distal' zone. These zones held true for the distribution of cells near the endosteum as well (Fig. 3d). These data cumulatively suggested that there are spatial hotspots of associations within the greater haematopoietic microenvironment and allowed us to define the criteria for associations of haematopoietic cells with the niche.

These spatial criteria were then imposed on the trace of the cell tracked in Fig. 3a; this analysis showed that the cell was initially in contact with the vasculature, followed by a proximal interaction with the vasculature and finally a proximal interaction with the endosteal region (Fig. 3e). We added functionality to our software, which enabled us to identify, classify and quantify these interactions automatically (see Methods). Using this tool, we found that the cells we tracked had 95 periods of interaction with either the vascular or endosteal regions and 24 periods of no interaction (that is, classified as distal to both regions) as a group. It is likely that other niche cells within the overall microenvironment can also serve as hotspots for associations and may be intermingled in the ‘proximal' and ‘distal' zones.

**Figure 4:**
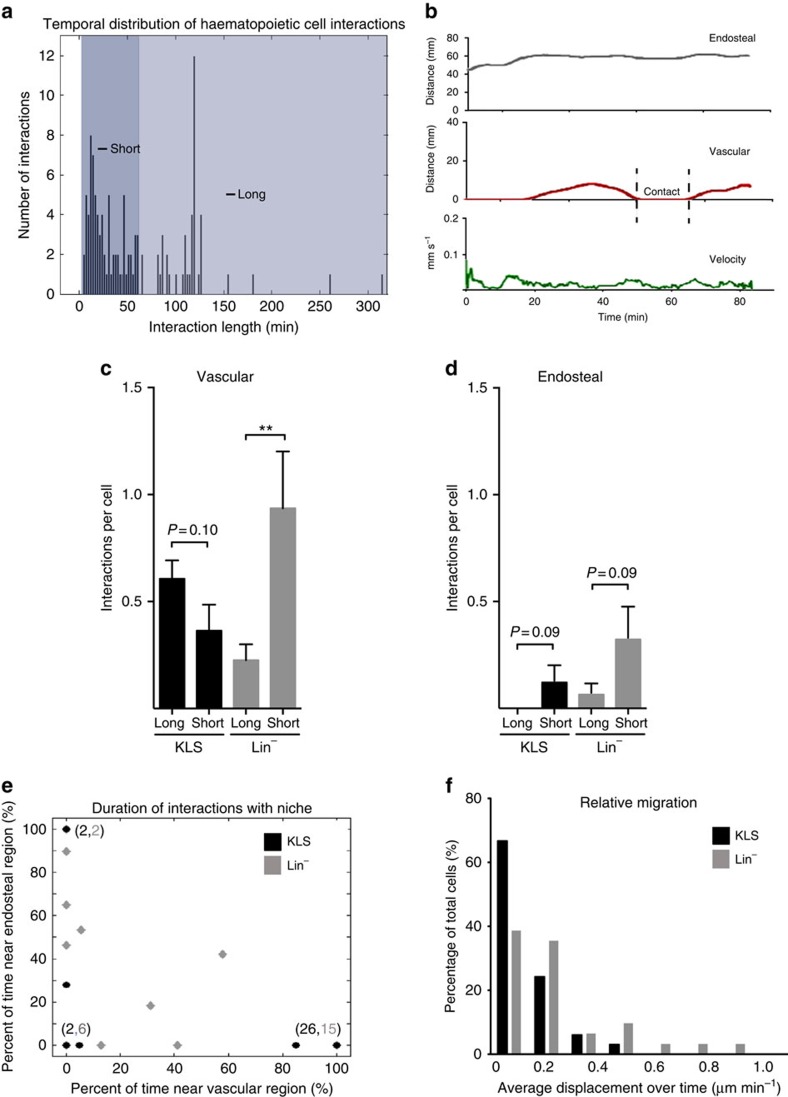
Comparative temporal dynamics of haematopoietic cells. (**a**) Histogram showing duration of all the interactions (instances where cells were in contact with or proximal to a given region) calculated by this software. Two categories of interaction duration were identified: short (<60 min) and long (>60 min). (**b**) Cell trace depicting a short contact with the vascular region. Distance to the endosteal (grey line) and vascular (red line) regions as well as cell velocity (green line) for a representative cell is shown, highlighting a short contact interaction (dashed gates) with the vascular region. (**c**–**f**) Categorization of the interactions of KLS (black) and Lin^−^ (grey) cells by region and duration. (**c**) The incidence of interactions per cell found in the vascular region, sorted by duration. *P*=0.10 for KLS (*n*=33 cells) and **P=0.0126 for Lin^−^ (*n*=31 cells) by Student's *t*-test. (**d**) The incidence of interactions per cell found in the endosteal region, sorted by duration. *P*=0.09 for KLS (*n*=33 cells) and *P*=0.09 for Lin^−^ (*n*=31 cells) by Student's *t*-test. Data represented as mean+s.e.m. (**e**) For each cell, the fraction of observation time (shown as per cent) spent near (<25 μm; in contact or proximal to) the vascular (*x* axis) and endosteal (*y* axis) regions is plotted as a single point. Point overlap is indicated in the parentheses, where the first number represents KLS (black circle) and the second number Lin^−^ (grey diamond) cells that fall in that point. (**f**) Histogram containing the normalized average displacement for KLS and Lin^−^ cells, where displacement is defined as the distance between a current cell position and its first recorded position, and the displacement is normalized by the total time a given cell was observed.

Interestingly, the duration of cell interactions varied from about 4 min to over 5 h. Of the cells we tracked, 7% moved through the blood vessels and 12% moved through the microenvironment in under 2 min (Fig. 3f); detailed measurements were thus extracted from the rest of the transplanted cells (*n*=95). On the basis of the distribution of duration of cell interactions, interactions could be categorized into two groups: the cluster of interactions lasting <60 min were termed ‘short'; and interactions >60 min were termed ‘long' (Fig. 4a). In the cellular trace used as an example in Fig. 4b, imposing such temporal criteria shows that the cell tracked in this case made one short contact with the vasculature lasting <30 min and remained distal to the endosteal niche at all times.

### Comparative dynamics

The ability to define interactions in terms of space and time provided suitable metrics for quantitatively comparing interactions made by distinct groups of cells. Using this approach, we compared the dynamics of transplanted haematopoietic cells at different stages of differentiation in a normal environment. Specifically, we compared the behaviour of three cell populations: (1) a stem cell-enriched population using sorted c-Kit^+^ Lin^−^ Sca-1^+^ (KLS), (2) a progenitor cell-enriched population using a lineage depletion (Lin^−^) and (3) a fully differentiated lineage-positive fraction (Lin^+^) isolated from the bone marrow. This comparison revealed marked temporal and spatial differences in the interactions of mature and immature haematopoietic cells with their microenvironment. As shown in Fig. 4c, KLS cells made approximately threefold more long interactions per cell with the vascular niche than Lin^−^ progenitors cells, which made mostly short associations. In contrast, both Lin^−^ progenitors and KLS cells made more short-term interactions with the endosteal niche than they did long-term associations (Fig. 4d). Moreover, haematopoietic cells were found to associate with vascular niches the majority of the time (Fig. 4e) and, consistent with their ability to interact with several microenvironmental elements, progenitor cells showed greater displacement from their origin over time (Fig. 4f).

**Figure 5:**
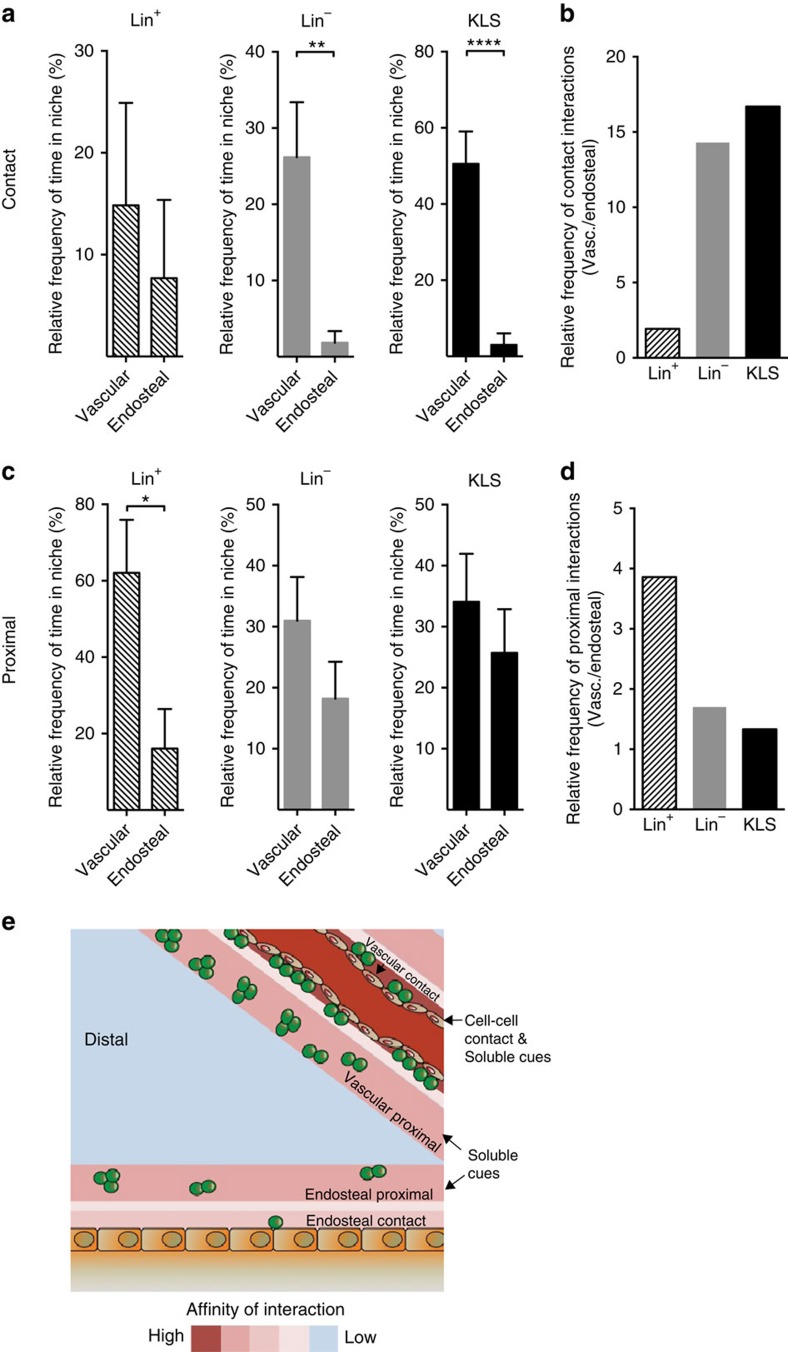
Comparative associations of haematopoietic cells with vascular and endosteal regions. (**a**) The relative fraction of time (shown as per cent) in contact with the vascular or endosteal niche. **P=0.0016 for Lin^−^. ****P<0.0001 for KLS by Student's *t*-test. Data represented as mean+s.e.m. (**b**) The relative contact frequency (vasculature/endosteal) of KLS (black), Lin^−^ (grey) and Lin^+^ (solid hatched line) cells. (**c**) The relative fraction of time in proximal interactions with the vascular or endosteal niche. *P=0.0137 for Lin^+^ by Student's *t*-test. Data represented as mean+s.e.m. (**d**) The relative proximal frequency (vascular/endosteal) of KLS (black), Lin^−^ (grey) and Lin^+^ (solid hatched line) cells. (**e**) Model showing microenvironmental regions enriched for progenitor cell (Lin^−^) associations in homeostasis as an example. Cells are preferentially in contact with or proximal to the vascular niche but mostly proximal to, rather than in contact with, the endosteal region. Warmer colours (red) identify areas with a high probability of associations and cooler colours (blue) identify areas with a low probability of associations.

Although the nature of the temporal interactions differed between KLS and Lin^−^ progenitor cells, both of these populations displayed highly significant spatial affinities for contacting the vascular area (Fig. 5a) compared with the endosteum. This preference was greatest in the most undifferentiated cells and decreased with differentiation (∼17-fold increase in affinity for KLS, 14-fold for Lin^−^ and 2-fold for Lin^+^ cells; Fig. 5b). Interestingly, cell interactions were more evenly distributed with the proximal domain of the vascular and endosteal niche (Fig. 5c,d). These data suggest a model where the programs that control lineage commitment and differentiation are closely linked to the spatial location and temporal interactions of cells within the niche, and that these molecular elements collectively ensure that the most immature cells receive cell–cell contact signals preferentially from the vascular endothelium, and soluble cues from both vascular and endosteal domains (Fig. 5e).

### Tracking endogenous haematopoietic cells with an Msi2 reporter

**Figure 6:**
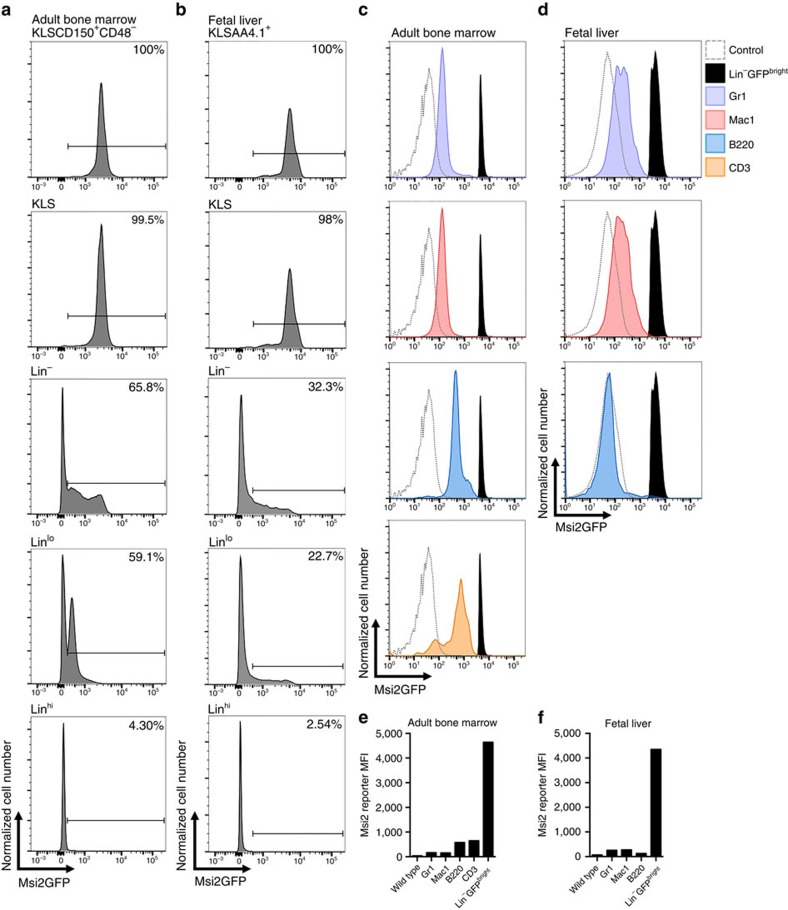
Msi2 reporter expression in stem/progenitor and differentiated haematopoietic cell populations. (**a**) Representative flow cytometry plots show histograms of Msi2GFP fluorescence intensity in KLSCD150^+^CD48^−^, KLS, Lin^−^, Lin^lo^ and Lin^hi^ cells from adult bone marrow. (**b**) Representative flow cytometry plots show histograms of Msi2GFP fluorescence intensity in KLSAA4.1^+^, KLS, Lin^−^, Lin^lo^ and Lin^hi^ cells derived from E15.5 fetal liver. (**c**) Representative flow cytometry plots showing GFP expression in control (wild type, non-reporter) bone marrow (light grey, dashed), Msi2GFP^bright^Lin^−^ cells (black) and differentiated cells from Msi2GFP reporter mouse (coloured). (**d**) Representative flow cytometry plots showing GFP expression in control (wild type, non-reporter) fetal liver cells (light grey, dashed), Msi2GFP^bright^Lin^−^ cells (black) and differentiated cells from Msi2GFP reporter mouse (coloured). (**e**) Quantification of mean fluorescence intensity (MFI) of Msi2GFP in control bone marrow (wild type, non-reporter), differentiated lineages and Msi2GFP^bright^Lin^−^ immature cells. (**f**) Quantification of MFI of Msi2GFP in control fetal liver, differentiated cells and Msi2GFP^bright^Lin^−^ undifferentiated cells.

To track endogenous haematopoietic stem and progenitor cells *in vivo*, we utilized the newly developed REM2 knock-in reporter mouse^18^. This reporter was created by knocking in the eGFP cassette into exon 1 of the Msi2 gene in frame with the ATG start codon. Because this is the first use of the Msi2GFP reporter mouse for imaging normal haematopoietic cells, we wanted to ensure that the disruption of one allele of the *Msi2* gene caused by insertion of the reporter cassette did not impair stem/progenitor cell function. Our experiments show that *Msi2*^*+/+*^ and *Msi2*^*+/GFP*^ long-term (LT)-HSCs have equivalent colony-forming ability *in vitro* (Supplementary Fig. 3a) as well as reconstitution ability *in vivo* (Supplementary Fig. 3b); further we could not detect any difference in the ability of *Msi2*^*+/+*^ and *Msi2*^*+/GFP*^ stem and progenitor cells to migrate towards chemokines indicating the heterozygous cells likely reflect normal haematopoietic cell behaviour (Supplementary Fig. 3c). In accordance with the known pattern of Msi2 expression, reporter expression was highest in immature stem/progenitor cells and decreased with differentiation and lineage commitment. Specifically, KLS cells, which contain both stem and progenitor cells, and KLSCD150^+^CD48^−^ cells, which represent highly purified stem cells, were 99.5% and 100% positive for reporter expression, respectively, and contained within the Msi2GFP^bright^ fraction (Fig. 6a). This pattern was also consistent during embryonic development: thus in the fetal liver, KLS cells and the more stem cell pure KLSAA4.1^+^ population were 98% and 100% positive for reporter expression, respectively (Fig. 6b), and expression dropped with differentiation. Overall, ∼90% of the Msi2GFP^bright^ population in the adult bone marrow, and 95% of the Msi2GFP^bright^ population in the E15.5 fetal liver, were Lineage^negative/lo^. Thus, Msi2GFP^bright^ expression identified a nearly pure Lineage^negative/lo^ population, one containing immature uncommitted cells that have not begun to express lineage markers and are thus not lineage committed. While there was some dim Msi2GFP expression in lineage committed cell populations in the bone marrow (B220, CD3, Gr-1 and Mac1), this expression was 7–28-fold dimmer than expression seen in immature cells, consistent with observations in the fetal liver (Fig. 6c–f).

**Figure 7:**
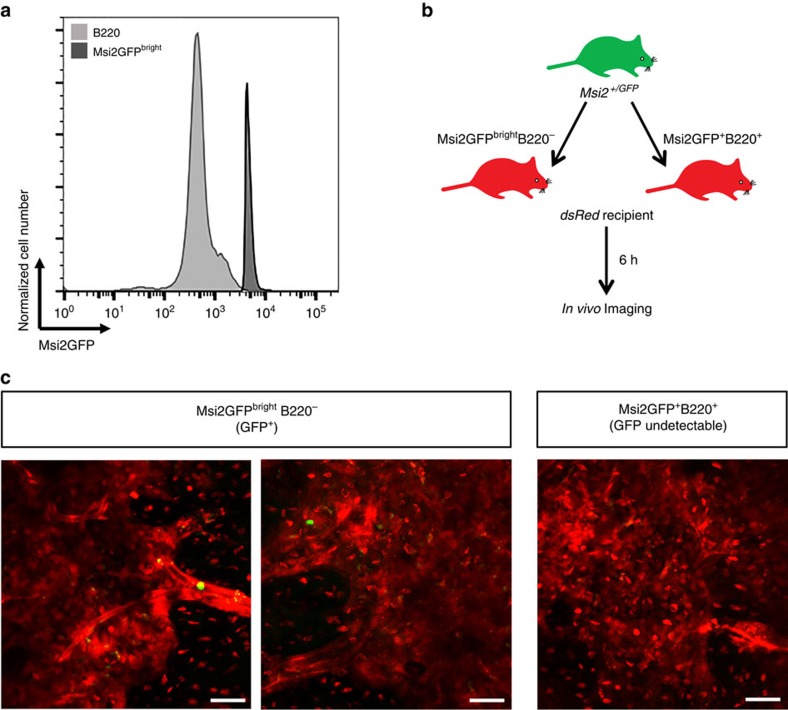
Imaging Msi2GFP^bright^ cells *in vivo* enables tracking of endogenous immature cells. (**a**) Representative flow cytometry plot showing Msi2 reporter fluorescence intensity in B220^+^ cells (light grey) and Msi2GFP^bright^ cells (dark grey). (**b**) Experimental design to image and compare fluorescence intensity of Msi2GFP^+^B220^+^ and Msi2GFP^bright^B220^−^ cells *in vivo*. (**c**) Representative images showing visible transplanted Msi2GFP^bright^B220^−^ cells after voltage-gated thresholding so Msi2GFP^+^B220^+^ cells were undetectable. Scale bar, 40 μm.

**Figure 8:**
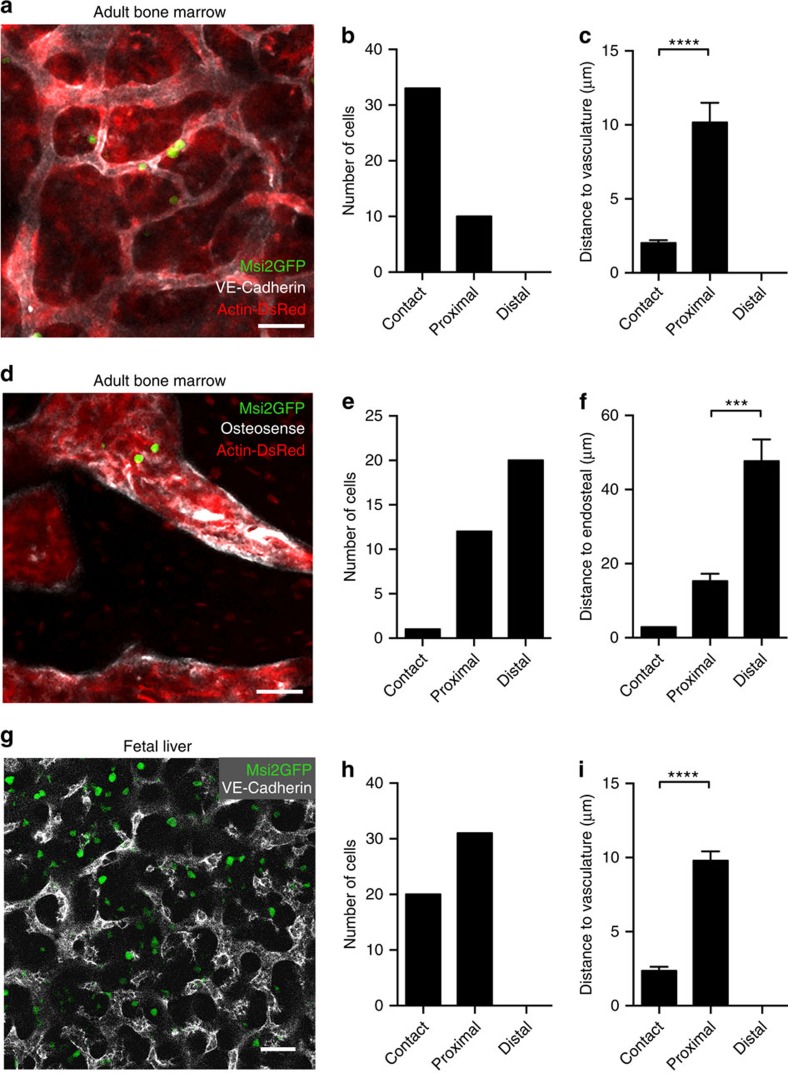
Comparative associations of endogenous immature haematopoietic cells with vascular and endosteal regions using Msi2 reporter. Representative image of Msi2GFP (green);dsRed (red) mice showing Msi2GFP^bright^ cells localized near vasculature (**a**) (white) and endosteum (**d**) (white). Scale bar, 40 μm. (**b**) Quantification showing the incidence of Msi2GFP^bright^ cells localized in contact with, proximal or distal to vasculature (*n*=43 cells from five mice). (**c**) Quantification of the mean distance to vasculature within contact, proximal and distal regions. ****P<0.0001 (*n*=43 cells from five mice) by Student's *t*-test. (**e**) Quantification showing the incidence of Msi2GFP^bright^ cells localized in contact with, proximal or distal to endosteum (*n*=33 cells from five mice). (**f**) Quantification of the mean distance to endosteum within contact, proximal and distal regions. ***P=0.0003 (*n*=33 cells from five mice) by Student's *t*-test. (**g**) Representative image of E15.5 Msi2GFP fetal liver showing Msi2GFP^bright^ cells (green) localized near vasculature (white). Scale bar, 40 μm. (**h**) Quantification showing the incidence of Msi2GFP^bright^ cells localized in contact with, proximal or distal to vasculature. (**i**) Quantification of the mean distance to vasculature within contact, proximal and distal regions. ****P<0.0001 (*n*=52 cells) by Student's *t*-test. Data represented as mean+s.e.m.

To set up our imaging parameters, we exploited the expression differential between differentiated B220^+^ cells and Msi2GFP^bright^ cells. Specifically, the fact that B220^+^ cells were on average eightfold dimmer (Fig. 7a), allowed us to threshold out the Msi2GFP^dim^ cells and visualize only the Msi2GFP^bright^ cells (Fig. 7b,c). The settings used were determined by transplanting Msi2GFP^bright^ and B220^+^ (Msi2GFP^dim^) cells into separate recipients and defining the voltage gates at which the B220^+^ cells were undetectable. To facilitate these studies, the Msi2GFP reporter mouse was crossed with a dsRed mouse to provide a counterlabel. Interestingly, all of the Msi2GFP^bright^ cells were localized in contact or proximally to the vasculature, with the majority being in contact with the vascular cells (Fig. 8a–c). In contrast, the majority of Msi2GFP^bright^ cells observed were localized distally from the endosteum (Fig. 8d–f). In addition to the spatial analysis, we also dynamically tracked Msi2GFP^bright^ cells over time. A representative Msi2GFP^bright^ cell is shown in contact with the vasculature (Supplementary Fig. 4a). When tracked relative to the vasculature, there was no change in the distribution of the distance over the time tracked (Supplementary Fig. 4b). Consistent with this pattern, none of the Msi2GFP^bright^ cells moved significantly enough to change the type of association (contact, proximal or distal) they had with the microenvironment. We also examined the spatial interactions of endogenous immature haematopoietic cells within the fetal liver; here Msi2GFP^bright^ cells associate with the vasculature via an even distribution of contact and proximal interactions (Fig. 8g–i). Importantly, these data are consistent with the interactions of transplanted immature cell populations, emphasizing the physiological relevance of our findings that native haematopoietic stem and progenitor cells are particularly dependent on niches set up by vascular domain.

## Discussion

The approach we report provides a new framework for integrating very high-resolution long-term *in vivo* imaging with a high-throughput computational analysis (Table 1). The ability to use confocal microscopy makes this approach to real-time imaging significantly more accessible and provides far greater multichannel resolution relative to existing two-photon *in vivo* microscopy^15^,^16^. This spatial clarity allowed visualization of fundamental biological processes such as cell migration, division, extravasation and intravasation. Imaging of both transplanted as well as endogenous haematopoietic cells from a Msi2GFP knock-in reporter mouse revealed that haematopoietic stem/progenitor cells are generally localized in contact with the vasculature, but distally from bone. Finally, we report a tool that enables high-throughput computational analysis of the ‘big data' generated from *in vivo* imaging. To our knowledge, a method to reliably automate spatiotemporal information from large volumes of imaging data does not exist at the current time; thus, the publicly available MATLAB program we have developed could be widely applicable, and thus a critical and novel contribution to the field.

The combination of the imaging resolution with the computational capacity provided, for the first time, quantified information about the activity of single cells in space and time *in vivo*. We analysed two microenvironmental elements implicated in haematopoiesis^2^,^3^,^4^,^6^,^7^,^8^,^9^,^10^,^11^,^12^ and show that immature haematopoietic cells appear to interact with each in distinct ways, with preferential contact made with vascular domains, and equivalent levels of proximal associations made with vascular and endosteal domains. In the long term, overlaying the base spatiotemporal map with a map of molecular reporter activity may allow definition of the signals that are differentially activated in response to particular cell–niche associations. Our strategy could be further expanded to study the dynamics of cell responses to injury, oncogenesis or inflammation. The microenvironmental changes in each of these contexts will be unique and need to be tracked; for example, after injury such as chemotherapy or radiation massive degeneration of microenvironmental structures occurs (Supplementary Fig. 5), indicating that associations may change markedly in regeneration. The temporal resolution achieved could be useful in gaining insight into the dynamics of haematopoietic cells in biological processes such as regeneration that unfold over time.

Analysing large amounts of image data has become a critical bottleneck in the discovery process^26^. To resolve this, we developed software that allows efficient quantitative characterization of each cell in terms of its position, motion and proximity to important niches. Approximately 41,968 frames were analysed in 20 min and required a total manual input time of ∼3.5 h. To compare it with fully manual analysis, we estimated that a person would need at least 30–60 s per frame to measure the distance between a cell and each microenvironmental domain, and a total of 350–700 h, indicating the software decreased manual work by 100-fold. The fact that the software provides a marked advantage over manual processing suggests it can serve as a more general high-throughput tool for spatiotemporal analysis of *in vivo* imaging data. The software also allowed calculation of the velocity of blood cells moving both within the bone marrow or flowing through a blood vessel. This capability could be useful, for example, in defining how closely ‘induced' blood cells generated from directed differentiation strategies resemble ‘naturally born' blood cells. In fact as the field of regenerative medicine matures, it is intriguing to speculate that this type of tool could prove important for *in vivo* assessment of cells derived from directed differentiation methods before clinical use^27^.

Our imaging analysis identified both spatial and temporal differences in the interactions of haematopoietic cells with vascular and endosteal regions in homeostasis (Supplementary Movie 10). Further, it revealed that the dynamic behaviour of haematopoietic cells *in vivo* is directly related to their differentiation state. Thus, haematopoietic stem/progenitor cell-enriched fractions have far higher spatial and temporal affinity for vascular domains, whereas differentiated cells shuttle more frequently between the vascular and endosteal domains, and exhibit less stable interactions. This suggests that immature cells are more dependent on the niches they are part of and thus retained more readily, and that this dependence diminishes with maturation, allowing cells to leave. In the longer term, the introduction of additional niche markers as well as other cell types could easily be accommodated by these techniques and allow the development of a comprehensive dynamic atlas of haematopoietic cell interactions within the bone marrow microenvironment.

The Msi2GFP reporter mouse is an exciting tool that has enabled us to dynamically track endogenous immature haematopoietic cells both temporally and spatially. Haematopoietic stem/progenitor cells in Msi2 reporter mice were largely localized in contact with the vasculature, and distally from the endosteum, suggesting that the vascular niche is particularly supportive of these undifferentiated cells in this context as well. Interestingly, the association of Msi2GFP^bright^ cells with the vascular domain was highly stable, with almost all encounters scored as ‘long' interactions. The dominance of stable long interactions was in contrast to the more temporally distributed interactions (long and short) recorded from transplanted stem/progenitor cells. While we have used the Msi2 reporters to establish a baseline for normal haematopoietic stem and progenitor cells, they could be useful in multiple contexts: for example, we have used this model to track heterogeneity within aggressive solid cancers, and find it enables successful identification of tumour propagating cells, and therapy resistance in pancreatic cancer^18^.

The work reported here highlights the power of visualizing tissues using high-resolution live microscopy to illuminate the bone marrow environment that is critical for the self-renewal and differentiation of haematopoietic stem and progenitor cells. The ability to observe different cell types simultaneously *in vivo* is a powerful tool for analysing and understanding the nature of processes such as regeneration or oncogenesis, where new regulators may be difficult to discover with static approaches alone. Because the principles of the imaging paradigm and computational analysis we developed can be applied broadly, our work also raises the exciting possibility that the use of this strategy may ultimately allow a dynamic view into an array of tissues and organs whose architecture and living physiology will be important areas of future investigation.

## Methods

### Animals and cell isolation

Haematopoietic stem and progenitor cells were isolated from 8–12-week-old Actin-GFP mice (Jackson Labs, Bar Harbor, ME, USA) as described^28^. Whole bone marrow was lineage depleted via magnetic-activated cell sorting (MACS; Miltenyi Biotec, Bergisch Gladbach, Germany) using an AutoMACS sorter (Miltenyi Biotec). Subsequent lineage-depleted cells were stained using phycoerythrin (PE)-conjugated antibodies CD3e, CD4, CD8, B220, CD11b, Gr-1 and Ter119 (eBiosciences), and sorted for Lin^+^ and Lin^−^ fractions. Analysis and cell sorting were carried out on a FACSVantage sorter (Becton Dickinson, Mountain View, CA, USA) at the Duke Cancer Center Flow Cytometry Core Facility. A total of 1.5 × 10^6^ GFP^+^Lin^−^ progenitors were transplanted via retro-orbital sinus into p15 DsRed2 or 8-week-old mice (STOCK Tg(CAG-DsRed*MST)1Nagy/J, Jackson Labs). Mice were imaged between 1 and 12 h after adoptive transfer of GFP^+^Lin^+^ and GFP^+^Lin^−^ progenitors. For KLS cell isolation, whole bone marrow was enriched for c-Kit via MACS (Miltenyi Biotec) using an AutoMACS (Miltenyi Biotec). c-Kit-enriched cells were labelled for PE-conjugated antibodies for CD3e, CD4, CD8, B220, CD11b, Gr-1 and Ter119, APC-conjugated antibody c-Kit, and PE-Cy5-conjugated antibody for Sca-1 (eBiosciences). Analysis and cell sorting were carried out on a FACS AriaIII sorter (Becton Dickinson). A total of 1.5 × 10^6^ GFP^+^KLS cells were transplanted via retro-orbital sinus into 8-week-old mice (STOCK Tg(CAG-DsRed*MST)1Nagy/J, Jackson Labs). Mice were imaged 24 h after adoptive transfer of GFP^+^KLS cells. REM2 (Msi2^+/GFP^) reporter mice were generated by conventional gene targeting (Genoway, France). The eGFP cassette was knocked into exon 1 of the *Msi2* gene in frame with the ATG start codon^18^. Msi2GFP reporter mice imaged were between 3 and 8 weeks of age. Both male and female mice were used for the experimental purposes. All animal experiments were performed according to the protocols approved by the Duke University and University of California, San Diego, Institutional Animal Care and Use Committee.

### Mouse preparation and imaging

Mice were anaesthetized by intraperitoneal injection of ketamine and xylazine (100/20 mg kg^−1^). Once mice were unresponsive to pedal reflex, heads were wiped down with 70% ethanol and hair was removed using Nair Hair Remover lotion (Church & Dwight Co., Inc., Princeton, NJ, USA). A midline incision was made using FST ToughCut Spring Scissors, 6-mm curved blade (Fine Science Tools Inc., Foster City, CA, USA), and the skin was removed to expose the calvarium. For inverted confocal microscopy using younger mice, the calvarium was kept moist with Aqua Poly/Mount (Polysciences, Inc., Warrington, PA, USA) during the imaging session. Mice were inverted and secured onto a custom microscope rig by placing a rubber band (size 10) through the bit of the mouth and observed through a 22 × 22-mm coverslip (VWR International, West Chester, PA, USA). Mice were immediately taken to the confocal microscope for imaging and were kept under anaesthesia using 1–3% isofluorane gas mixed with oxygen. For upright confocal microscopy, mice were placed in a mouse/neonatal rat stereotactic holder (Stoelting Co., Wood Dale, IL, USA), calvarium was exposed as described above and tissue was kept moist using 1 × PBS (Gibco).

### Microscopy

Images were acquired by Leica LAS AF 1.8.2 software with either an inverted Leica SP5 confocal system using a Leica DMI6000CS microscope or an upright Leica SP5 2 confocal system using a Leica DM 6,000 CFS microscope. Using the inverted microscope, images were acquired using a × 10 Leica Plan Apochromat objective with 0.40 numerical aperture for quantification and a × 20 Leica Plan Apochromat objective with 0.70 numerical aperture. Using the upright microscope, images were acquired using an HCX APO L20x objective with a 1.0 numerical aperture for still images and subsequent movies. Imaging of calvarium ranged from 60 to 100 μm. CFP (excitation 458 nm and emission 463–500 nm), GFP (excitation 488 nm, emission 493–556 nm) and DsRed2 (excitation 561 nm, emission 566–650 nm) were excited with an Argon/2 (458, 477, 488, 496 and 514 nm) and Diode pumped solid-state (561 nm) laser, respectively. The power used for dsRed visualization was 8–12% of the appropriate laser. Images were continuously captured in 1,024 × 1,024 or 1,024 × 512 format, with line averaging of 4 (∼10 or 5 s per scan, respectively) for up to 8 h. Multicolour imaging for CFP and GFP were captured sequentially.

### Methylcellulose colony formation assay

LT-HSCs (KLSCD150^+^CD48^−^) were isolated by FACS from bone marrow. Cells were plated in methylcellulose medium (Methocult GF M3434 from StemCell Technologies). Colonies were counted 7 days after plating.

### *In vivo* transplantation assay

A total of 500 LT-HSCs isolated from bone marrow of mice expressing CD45.2 were transplanted into lethally irradiated (9.5 Gy) CD45.1 recipient mice with 3 × 10^5^ Sca-1-depleted CD45.1 bone marrow cells. Peripheral blood of recipient mice was collected at 4 weeks after transplantation.

### Chemotaxis assay

Directed cell migration towards SDF1 was analysed *in vitro*. Cells were kept in *X-VIVO* media (Lonza) and 600 μl *X-VIVO* media supplemented with 50 ng ml^−1^ of SDF1 was added to the lower chamber of the transwell (Costar, pore size 5 μm, 3,421). A total of 75,000 cells were loaded into the upper chamber and allowed to migrate for 18 h at 37 °C in a humidified CO_2_ incubator. After incubation, migrated cells were collected from the lower chamber and counted.

### *Ex vivo* fetal liver preparation and microscopy

Mouse embryonic fetal livers were dissected at stage E15.5 from timed mating females. Fetal livers were incubated on ice with fluorescently conjugated antibodies for VE-Cadherin (eBiosciences). Fetal livers were plated for imaging in 1.5% low melting agarose (Sigma) with *X-VIVO* media (Lonza) and 10% fetal bovine serum. Cultures were maintained at 37 °C and 5% CO_2_ using a Heating Insert P Lab-Tek S1 with an Incubator PM S1 (Zeiss). Images were acquired using an Axio Observer Z1 microscope with the LSM 700 scanning module (Zeiss).

### *In vivo* probe administration

Angiosense 680 and OsteoSense 680 *in vivo* imaging probes (VisEn, Bedford, MA, USA) were administered at a concentration of 2 nM in 150 μl per mouse, and imaged within 30 min (Angiosense) or at least 24 h (OsteoSense) after administration. Both products were excited using a HeNe 633 laser and emission was collected from 650 to 725 nm. Antibodies conjugated to AlexaFluor 647 for VE-cadherin (eBiosciences) and F4/80 (eBiosciences) were administered at a concentration of 10 μg diluted in 100 μl, 15 min before imaging. All products were excited using a HeNe 633 laser and emission was collected from 650 to 725 nm.

### Quantitative analysis

Images were analysed using Volocity Software (Improvision, a PerkinElmer Company, Coventry, England). Red and green channel noise was optimized using the fine filter, and image intensity gamma was used to reduce background within the green channel. For GFP quantification, × 10 *z*-stacks (30 *z*-planes for 120 μm) were analysed. Briefly, objects were filtered by intensity and size, and the sum of the isovolume (μm^3^) measurements was compared between the mice. Image enhancement and quantification parameters were identical between paired animals for each experiment. Movies were made using Volocity software and exported to view as AVI movies at 15 frames per second, and compressed using Microsoft Video 1 compression. Supplementary Movie 2 rolling movie frame rate was reduced to 5 frames per second.

### High-throughput imaging analysis

All image processing and object tracking were performed in MATLAB (R2010b). Supplementary Movie 11 is a representative movie that was used for the analysis. First, the movies were corrected for lateral (*xy*) drift by examining the cross-correlation between the first frame and every subsequent frame. Images labelled with cells were median filtered with a window size of 5 pixels, and then thresholded with a manual cutoff. Each candidate cell object was identified and the centroid calculated in each frame. Cells were tracked through time using particle-tracking software (http://physics.georgetown.edu/matlab/) and only tracks longer than 10 frames (∼100 s) were considered valid. The cells moved occasionally in three dimensions, briefly disappearing from the image for certain time points. In such cases, the position data for that time point was estimated by linear interpolation between the values obtained from the preceding and following images. As a final check, the quantified data (*x*,*y* positions for each cell at each time point—as shown in Supplementary Data 1) was superimposed on the image stacks, and the resulting movies were then subjected to a manual review to ensure that no errors were made in tracking. To classify cell locations as either vascular or endosteal, cell positions were compared with hand annotated images of vascular and endosteal regions, as shown in Supplementary Fig. 6. The minimum distance to a vascular and endosteal region was calculated for each position. To specify proximal and contact interactions, the distances to vascular and endosteal regions were compared across all data sets for all time points and distance cutoffs chosen appropriately. Interactions were classified as vascular or endosteal based on which region was closest, and contact and proximal interactions were decided with the previously described distance cutoffs. False interactions were suppressed in two ways: (1) contact and proximal distances were automatically adjusted by ±1 μm for each cell and (2) adjacent transient interactions (<200 s) were merged together. Finally, the track and interaction graph for each cell was verified by manual inspection. MATLAB scripts and extracted data files have been provided as Supplementary Software.

### Data availability

The data that support the findings of this study are available from the corresponding author on request.

## Additional information

**How to cite this article:** Koechlein, C. S. *et al*. High-resolution imaging and computational analysis of haematopoietic cell dynamics *in vivo*. *Nat. Commun.* 7:12169 doi: 10.1038/ncomms12169 (2016).

## Supplementary Material

Supplementary Figures Supplementary Figures 1-6

Supplementary Data 1 Raw Data for individual KLS cell Trace. An example of the raw data produced by tracking an individual KLSGFP^+^ cell. An x and y coordinate is listed for each timepoint analyzed (t).

Supplementary Movie 1 Bone marrow microenvironment. Representative movie of transplanted cells in bone marrow of dsRed^+^ transgenic mouse. LinGFP^+^ cell (arrow) is observed in the microenvironment. Time stamp is located in the upper right hand corner of the movie in approximately 10.3-second intervals. Shown is 01:48:57 (hours:minutes:seconds) of a 06:01:23 movie. Scale bar = 100μm.

Supplementary Movie 2 Cell rolling along vessel wall. LinGFP^+^ cell (arrow) rolling along endothelium from time 00:00 to 02:04. LinGFP^+^ cell (arrow) migrating in the stroma from 02:14 to 05:00. Duration of movie, 05:41, minutes:seconds. Scale bar = 80μm.

Supplementary Movie 3 Cell division. Representative movie of a LinGFP^+^ cell undergoing division (arrow) in real-time during a 00:25:02 (hours:minutes:seconds) time period. Scale bar = 80μm.

Supplementary Movie 4 Multicolor imaging. Representative 20x z-step where CFP^+^ and GFP^+^ whole bone morrow was transplanted into a dsRed recipient. Each zstep is approximately 5μm for a total of 60μm of skull depth. Scale bar = 80μm.

Supplementary Movie 5 Microdomain analysis with CFP/TNR transgenic mouse. Representative movie of dsRed^+^ whole bone marrow transplanted into CFP^+^ TNR^+^ recipients (blue and green respectively). Duration of movie is 02:39:42:634 (hours:minutes:seconds:milliseconds) Scale bar = 80μm.

Supplementary Movie 6 Analysis of interactions of hematopoietic cells with the vasculature. Representative movie of GFP^+^ Lin- cells (green) transplanted in dsRed recipient mice co-injected with anti-VE-cadherin antibody to mark vascular endothelial cells (white). Duration of movie is 01:27:09:526 (hours:minutes:seconds:milliseconds). Scale bar = 70μm.

Supplementary Movie 7 Analysis of interactions of hematopoietic cells with the endosteal surface. Representative movie of GFP^+^ Lin- cells (green) transplanted in dsRed recipient mice co-injected with Osteosense probe to mark the endosteal surface (white). Duration of movie is 01:29:05:706 (hours:minutes:seconds:milliseconds). Scale bar = 70μm.

Supplementary Movie 8 Analysis of tissue macrophages in the bone marrow microenvironment. Representative movie of a dsRed^+^ mouse labeled with anti-F4/80 monoclonal antibody (white) to mark tissue macrophages in the bone marrow microenvironment. Duration of movie is 01:32:53:686 (hours:minutes:seconds:milliseconds) Scale bar = 140μm.

Supplementary Movie 9 In vivo trace of a single cell. Representative movie illustrating migration of LinGFP^+^ cells over time. The boundary of the vascular domain is marked in red and the endosteal domain is outlined in gray.

Supplementary Movie 10 Visualizing Stem Cell Dynamics in the Bone Marrow. Animated movie projection of hematopoietic cell dynamics in vivo, highlights how large amounts of visual information from our imaging strategy can be effectively processed through our computational analysis approach. All three zones of interaction (contact, proximal and distal) with bone or vessels are marked. Stem cells (blue) have a greater frequency of long-term contact interactions with vasculature compared to bone. Progenitor cells (green) have greater frequency of short-term contact interactions with vasculature.

Supplementary Movie 11 Raw Data for individual KLS cell Trace. An example of the raw data produced by tracking an individual KLSGFP^+^ cell. An x and y coordinate is listed for each timepoint analyzed (t).

Supplementary Software High-throughput computational method for single cell analysis of live imaging MATLAB scripts were used to analyze large volumes of live imaging data. This software is able to automatically track single hematopoietic cells over time to classify and quantify their spatiotemporal interactions within the native microenvironment. Both MATLAB scripts and extracted data files are provided.

## Figures and Tables

**Table 1:**
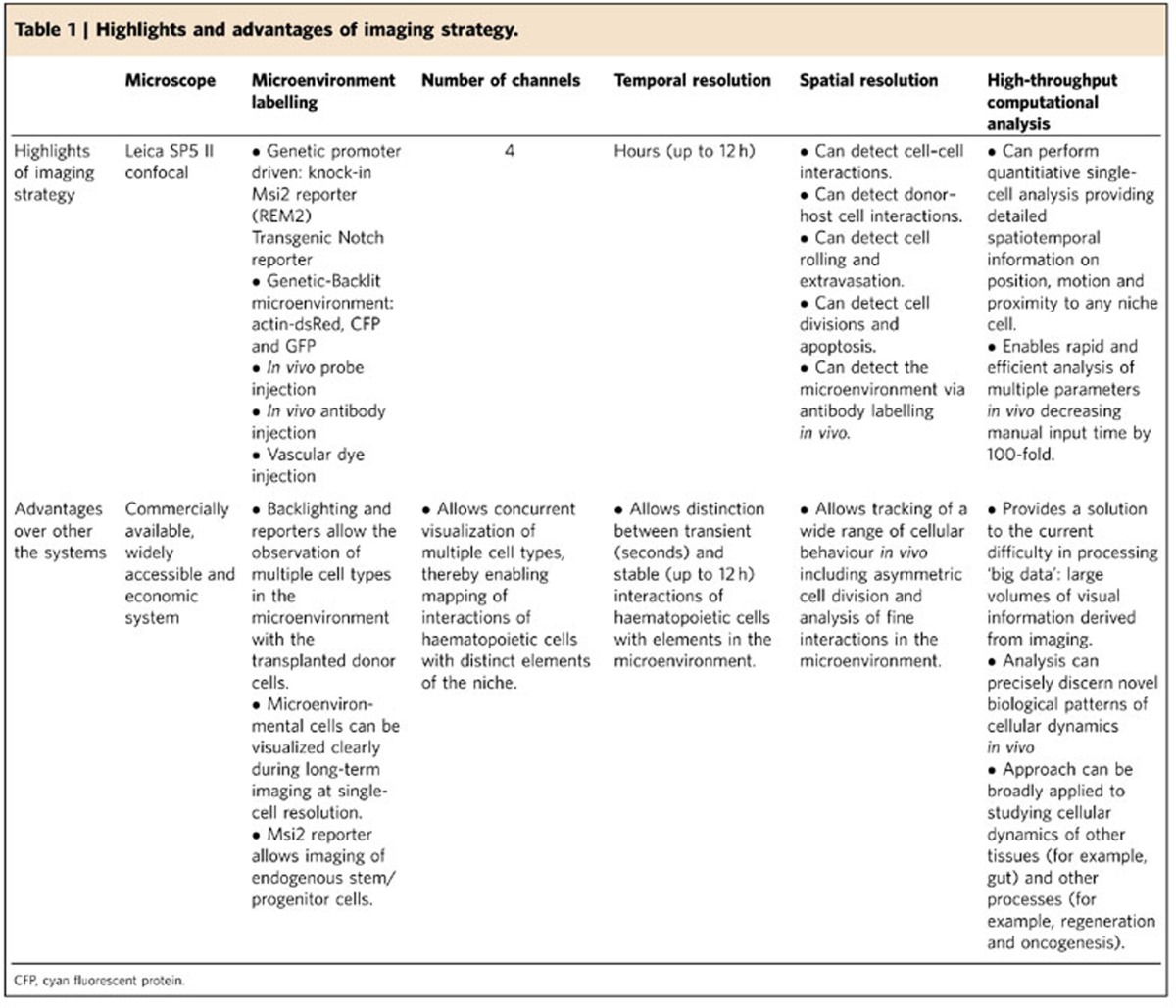
Highlights and advantages of imaging strategy.
