# Supplementary material for: High-resolution imaging and computational analysis of haematopoietic cell dynamics in vivo
Source: Nat Commun. 2016 Jul 18;7:12169. doi: 10.1038/ncomms12169 (PMC4960315; doi:10.1038/ncomms12169)
Supplement: Supplementary Figures — 1-6 [file ncomms12169-s1.pdf]

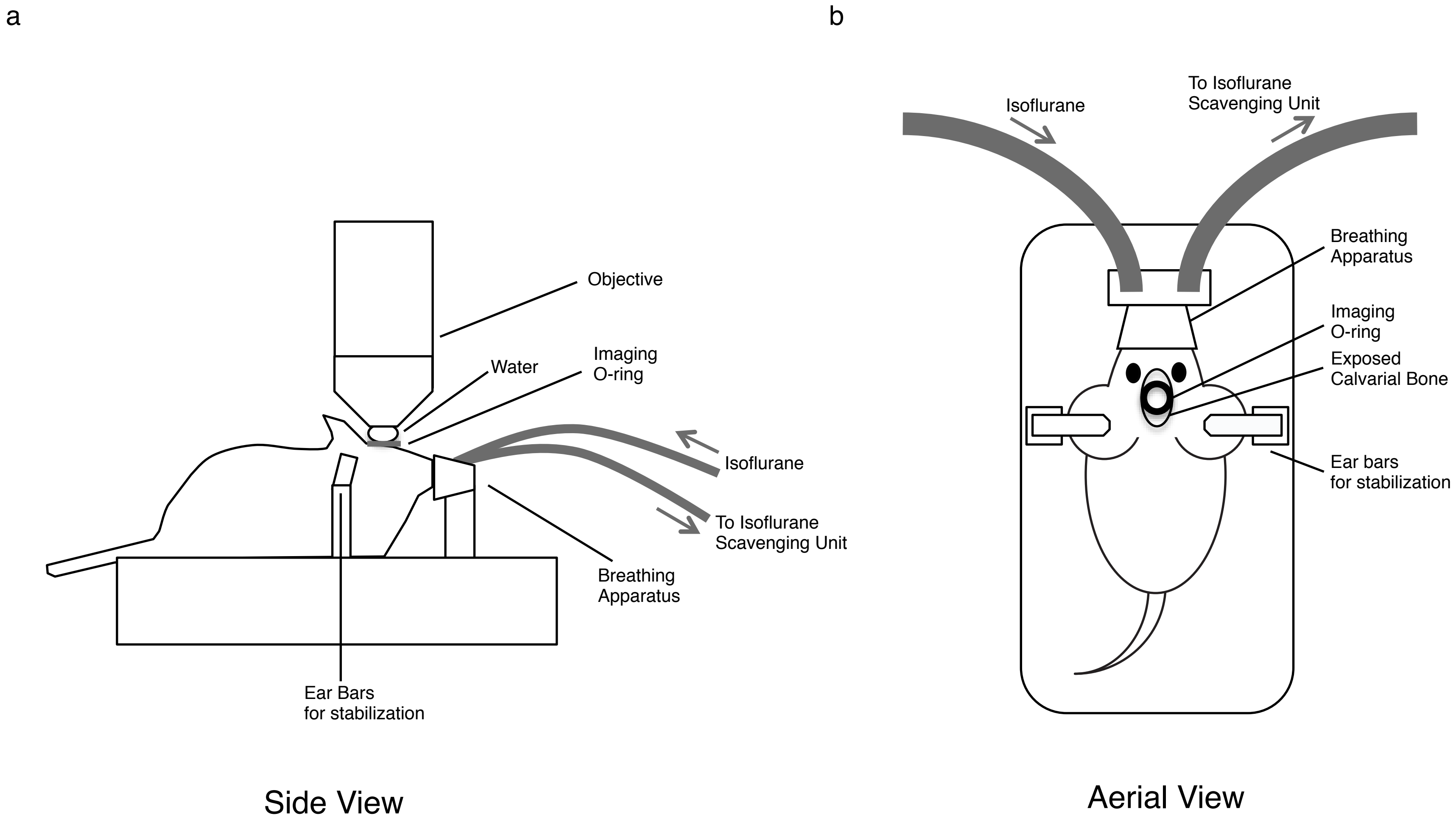

Supplementary Figure 1. Schematic of intravital mouse imaging system
(a) A side view depicting an anesthetized mouse placed in a stereotactic holder prepared for confocal microscopy. Ear bars stabilize the mouse in the holder. A breathing apparatus keeps the mouse under anesthesia with isoflurane gas. An imaging O ring is secured to exposed calvarial bone. Water is placed in the O ring, through which the objective is able to image the calvarium (b) An aerial view depicting an anesthetized mouse placed in a stereotactic holder prepared for confocal microscopy.

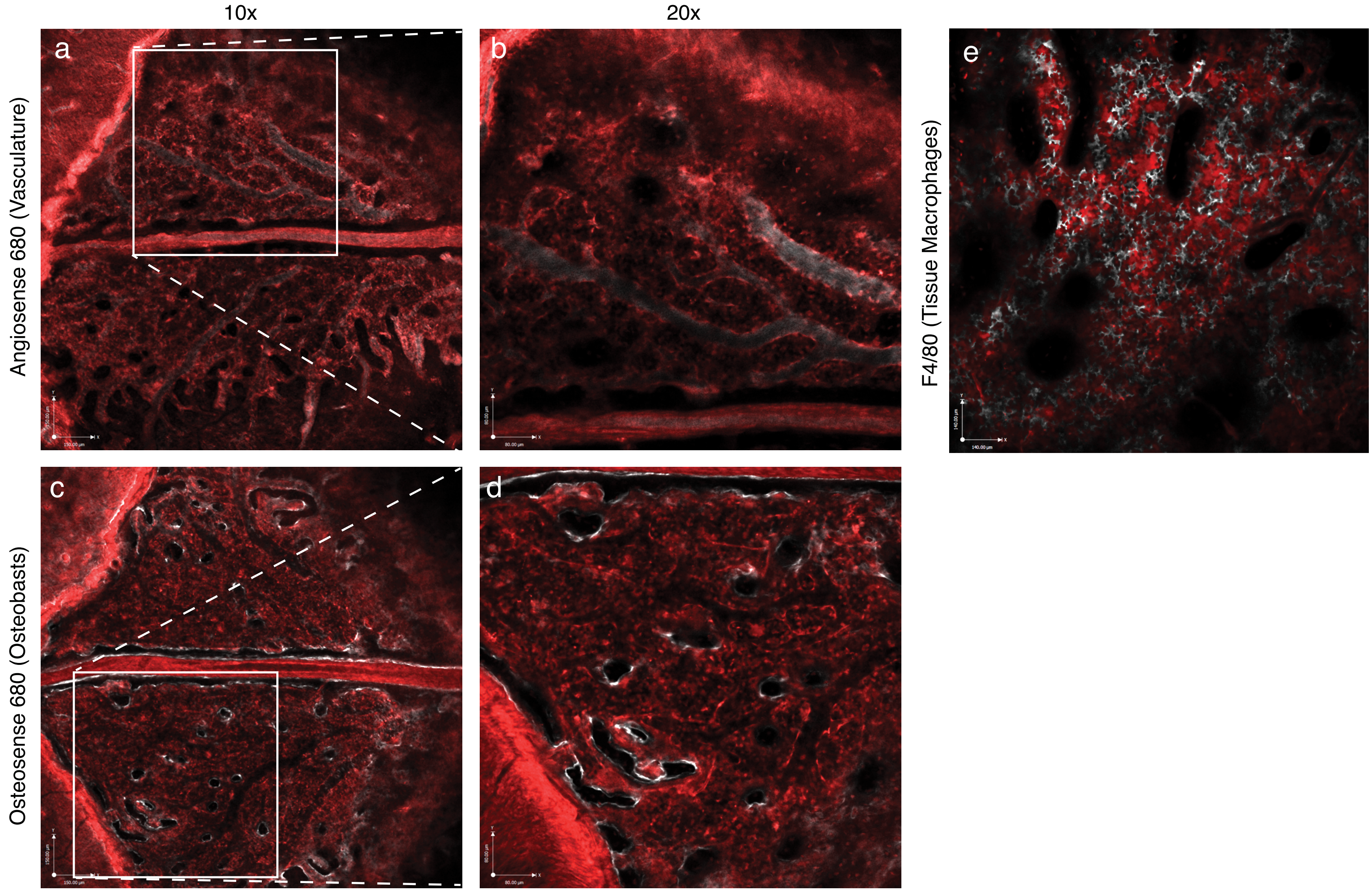

Supplementary Figure 2. Detection of vascular and endosteal surface in the bone marrow microenvironment
Representative 10x and 20x images of mice treated with Angiosense 680 probe (a and b), which marks the vasculature. Representative 10x and 20x image of mice treated with Osteosense 680 probe (c and d), which marks active osteoblasts. (e) Still image of dsRed recipient labeled in vivo with monoclonal antibody for tissue macrophage marker F4/80. See corresponding Supplementary Movie 8.

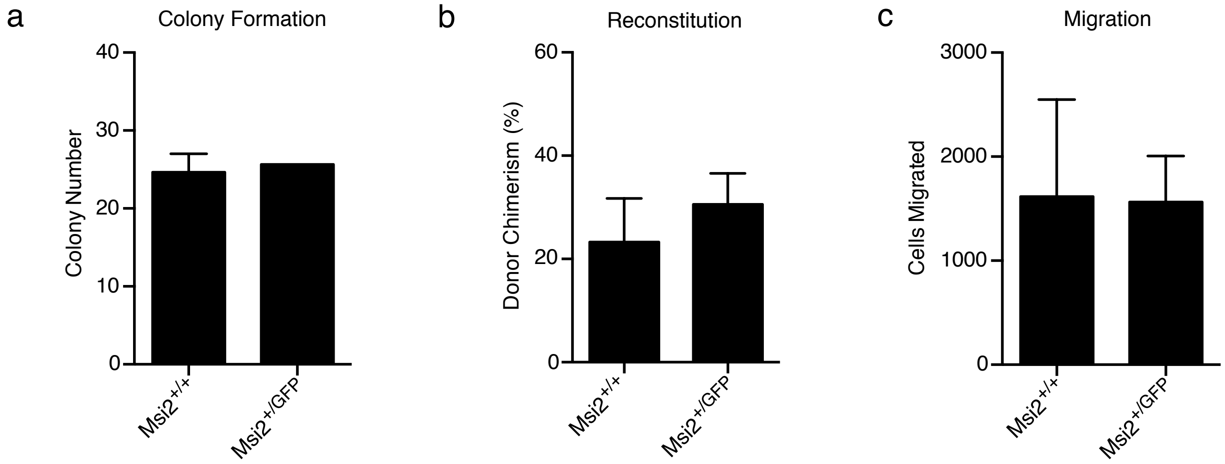

Supplementary Figure 3. Functional Analysis of Msi2GFP hematopoietic cells
(a) Number of colonies generated from Msi2$^{+/+}$ and Msi2$^{+/GFP}$ LT-HSCs (KLSCD150+CD48-) (Not significant by Student's t-test, n=3 technical replicates). (b) Average donor chimerism 4 weeks after transplantation (Not significant by Student's t-test, 3-5 mice per cohort). (c) Number of ckit+Lin- cells from Msi2$^{+/+}$ and Msi2$^{+/GFP}$ mice migrated in response to SDF1 (Not significant by Student's t-test, n=3 technical replicates). Data represented as mean+SEM.

a

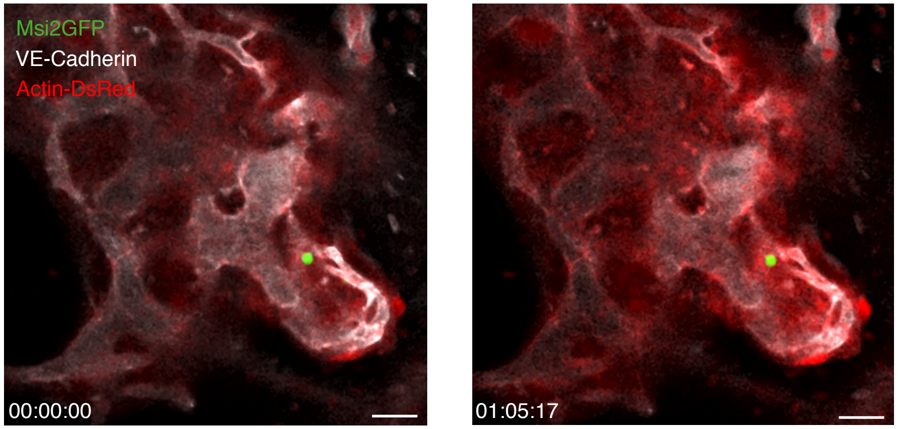

b

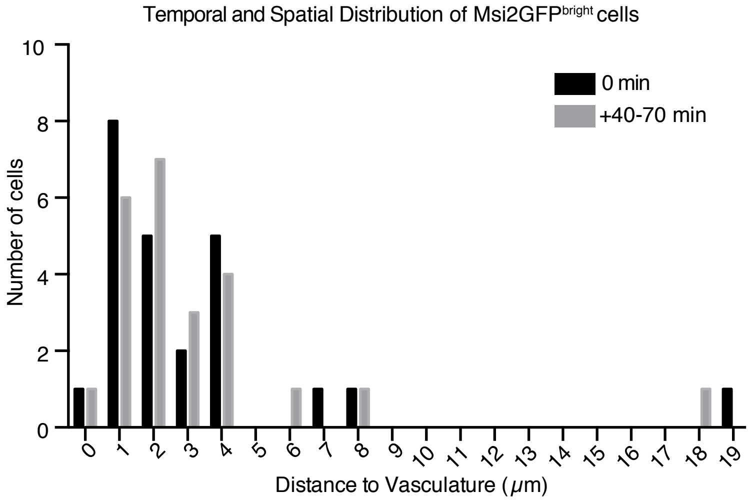

Supplementary Figure 4. Temporal Analysis of Msi2 reporter interactions with the niche
(a) Representative image of a Msi2GFP^{bright} cell (green) localized in contact with vasculature (white), shown are images taken at t=0 (left panel) and at endpoint t=01:05:17 (right panel). Scale bar = 40μm. (b) Histogram showing the distance of Msi2GFP^{bright} cells to vasculature at t=0 (black) and t=40-70min later (grey) (n=24 cells from 3 mice).

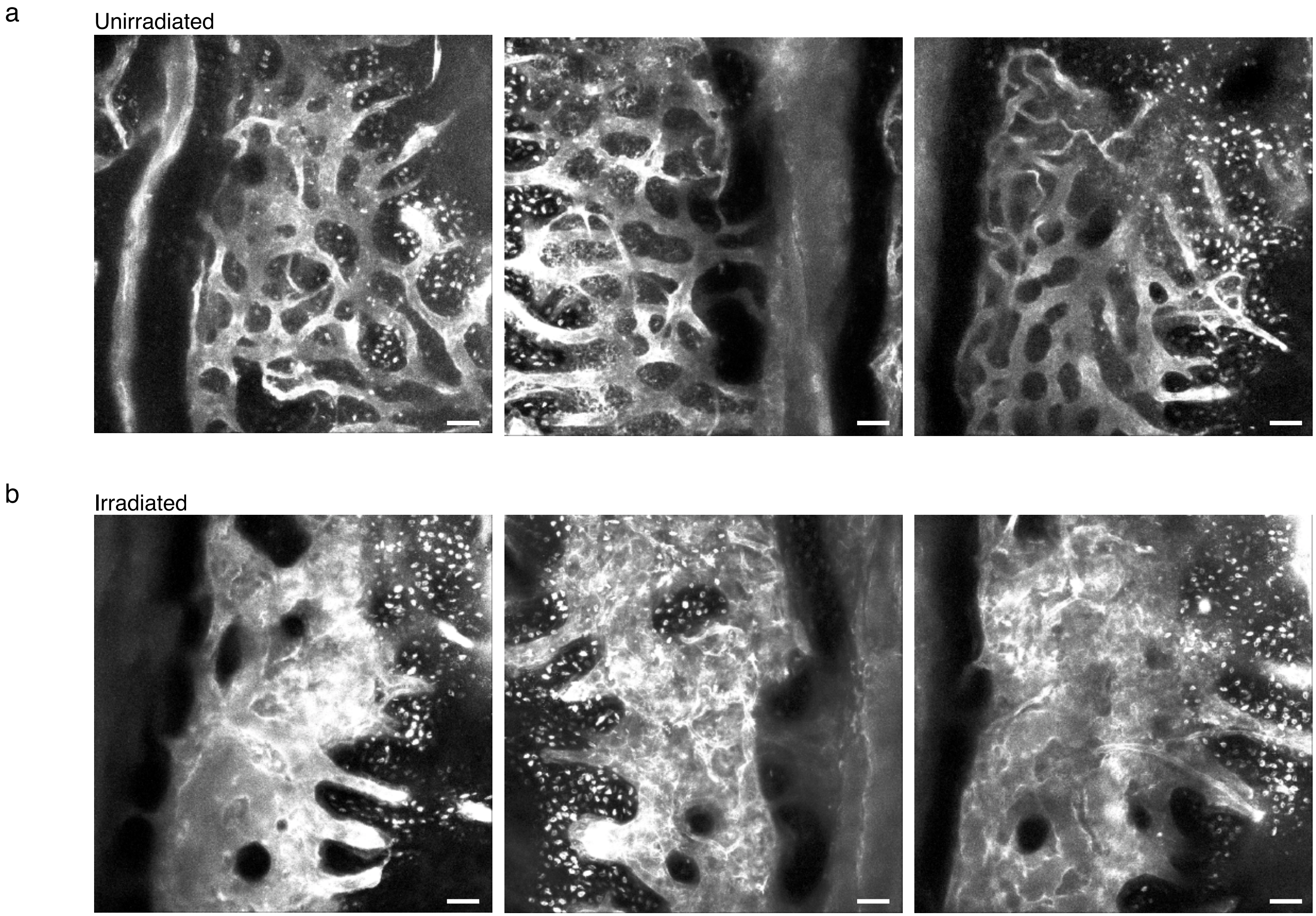

Supplementary Figure 5. Irradiation causes severe degradation of the bone marrow microenvironment
Representative images of bone marrow vasculature (white) in an unirradiated mouse (a) and a lethally irradiated (9.5Gy) mouse (b). Scale bar = 60 µm.

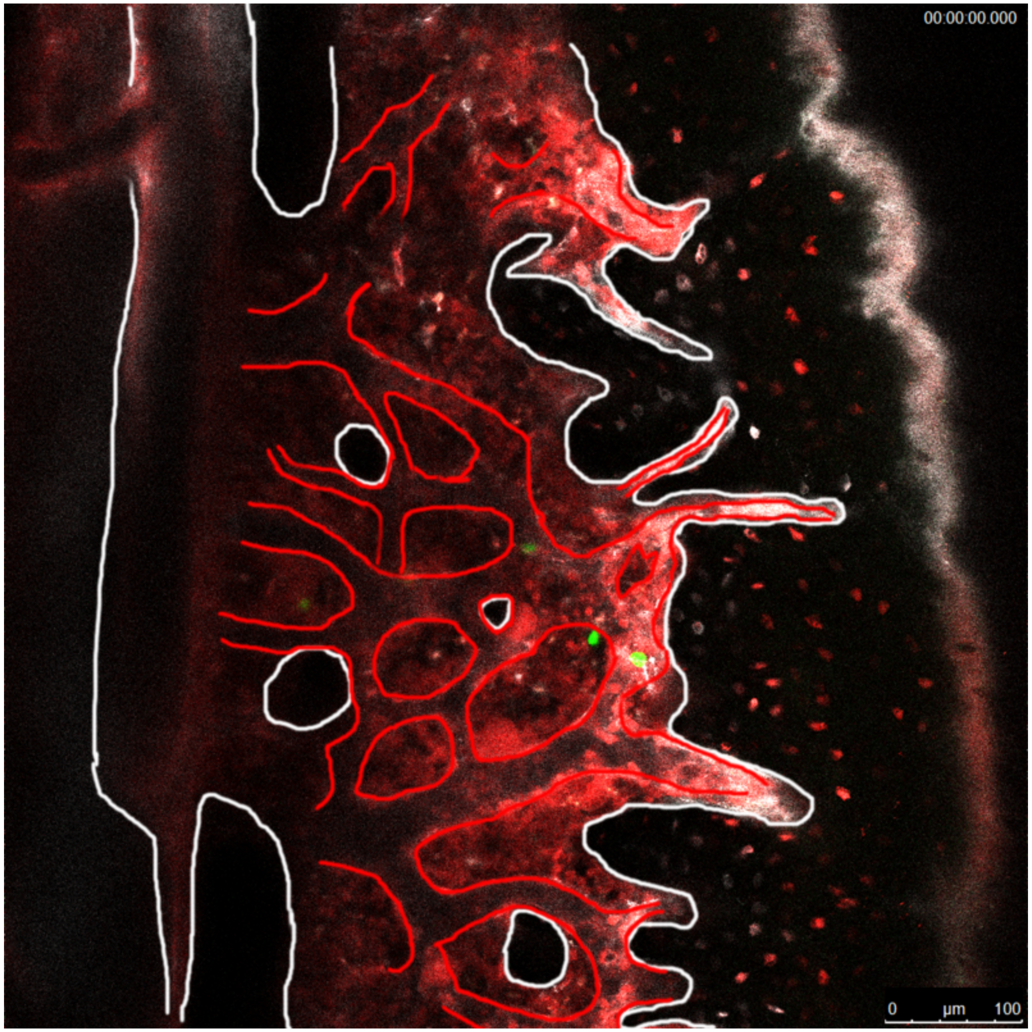

Supplementary Figure 6: Representative Image of Niche Domain Mapping
A representative frame from a video of KLSGFP+ cells transplanted into a DsRed mouse. The location of domains was traced manually (vasculature in red, endosteum in white). This trace is used by our software to determine the distance of cells of interest to microenvironmental domains. Scale bar = 100 µm.
